# The Odontogenic Keratocyst Conundrum- Is There an Answer?

**DOI:** 10.30699/ijp.2021.529691.2636

**Published:** 2021-08-09

**Authors:** B. Sabarinath, GB. Protyusha, S. Dhanarathna, Prakash Kaushik

**Affiliations:** 1 Department of Oral Pathology and Microbiology, Meenakshi Ammal Dental College and Hospital, Meenakshi Academy of Higher Education and Research, Chennai, India; 2 Oral and Maxillofacial Pathologist, Chennai, India; 3 Department of Orthodontics and Orofacial Orthopaedics, Meenakshi Ammal Dental College and Hospital, Meenakshi Academy of Higher Education and Research, Chennai, India

**Dear Editor**

Odontogenic keratocyst (OKC) is one of the controversial benign odontogenic pathologies in the maxillofacial region, which has undergone repeated conceptual and terminological changes since the time of inception in 1876 (1). This enigmatic developmental cyst is unique in terms of its peculiar nature and has garnered overwhelming attention from researchers all over the world. Since then, researchers have been trying to understand the nature and behavior of this dilemmatic lesion leading to repeated classifications and reclassifications. Previously classified as the developmental odontogenic cyst of the jaw by the World Health Organisation (WHO) in 1971 and 1992, OKC has been reclassified as a benign odontogenic neoplasm by the name of Keratocystic Odontogenic Tumor (KOT) in the third revision of WHO in 2005. However, in the latest WHO classification in 2017, KOT was reverted to the category of cysts (2). Therefore, the uncertainty about the designation of OKC as a cyst or a cystic neoplasm seems far from being resolved.

The OKC has always been a subject of interest to researchers and a conundrum to surgeons and pathologists due to its unique biological behavior, namely its aggressive and infiltrative nature, increased recurrence rate, and the association of multiple OKCs with Nevoid Basal Cell Carcinoma Syndrome (NBCCS). These were the pivotal reasons that prompted WHO to change the status of OKC from cyst to neoplasm as it was thought to reflect better the neoplastic nature of this lesion (3). The epithelial lining of OKC/KOT demonstrates higher levels of p53, Ki67, and PCNA immunoreactivity suggesting active cellular proliferation/differentiation in the pathogenesis of the lesion (4). In addition, the neoplastic nature of OKC/KOT could be attributed to the mutation of *PTCH1*, a tumor suppressor gene causing the misregulation of the Hedgehog signalling pathway (4). The recurrence rate of OKCs may have a range of 0%-100% which may result from a myriad of histopathological aspects of this lesion, including a thin friable lining epithelium with infolding elevating the chance of infiltration into the marrow spaces, the growth of new cysts from small satellite/ daughter cysts left behind during the surgical treatment, and active budding of the basal layer of the epithelial lining (5, 6). Although the biological behavior, recurrence rate, and PTCH mutation were the main reasons for the WHO consensus in 2005 to shift towards neoplasm, it was not unanimous owing to inadequate genetic and molecular evidence. The *PTCH* gene mutation was observed more in NBCCS cases than in sporadic OKCs. Moreover, *PTCH* gene mutations have been reported in other odontogenic cysts, such as orthokeratinized odontogenic cysts and dentigerous cysts (2).

The OKC has a morphology of a cyst denoting a pathological cavity and being referred to as a tumor, which necessarily means a swelling or neoplasm does not quite abide by the morphological definition of a tumor. Although the lesion has an aggressive nature with a high recurrence rate, it does not show neoplastic nature in terms of growth autonomy. It can resolve completely with simple marsupialization and decompression (2, 7, 8). These justifications may have been the cause of reclassification as a cyst by WHO in 2017. However, the nature of OKC/KOT continues to be the same as it was during the WHO (2005) classification, making the rationale of the reclassification questionable.

The dilemma concerning the classification and naming of this lesion simply shows the lack of understanding of this intriguing lesion despite advances in genetic and molecular studies. Although shuffling evolves with the current knowledge about the lesion at that time, what matters is the biological behavior of the lesion for adopting an appropriate treatment strategy. The perpetual confusion regarding the neoplastic nature of the cyst and its naming is due to inadequate evidence or a lack of comprehension of its nature among the researchers making it a never-ending debate. However, what requires attention and preferably a solution is the skepticism among the surgeons over the management of this lesion: should it be treated as a cyst or neoplasm? Nevertheless, it appears that only time has the answer as one can only wait eagerly until the following WHO classification of odontogenic cysts and tumors to know whether OKC will remain as a cyst or once again be rewarded with a new designation!

## Conflict of Interest

The authors declared no conflicts of interest.
